# Remarkable plasma-resistance performance by nanocrystalline Y_2_O_3_·MgO composite ceramics for semiconductor industry applications

**DOI:** 10.1038/s41598-021-89664-9

**Published:** 2021-05-13

**Authors:** Hyeon-Myeong Oh, Young-Jo Park, Ha-Neul Kim, Kundan Kumar, Jae-Woong Ko, Chae-Eon Lee, Hyun-Kwuon Lee

**Affiliations:** 1 Engineering Ceramics Department, Korea Institute of Material Science, 797 Changwondaero, Changwon, Gyeongnam 51508 Republic of Korea; 2 School of Advanced Materials Science and Engineering, Kumoh National Institute of Technology, Gumi, Gyeongbuk 39177 Republic of Korea

**Keywords:** Materials science, Nanoscience and technology

## Abstract

Motivated by recent finding of crystallographic-orientation-dependent etching behavior of sintered ceramics, the plasma resistance of nanocrystalline Y_2_O_3_-MgO composite ceramics (YM) was evaluated for the first time. We report a remarkably high plasma etching resistance of nanostructure YM surpassing the plasma resistance of commercially used transparent Y_2_O_3_ and MgAl_2_O_4_ ceramics. The pore-free YM ceramic with grain sizes of several hundred nm was fabricated by hot press sintering, enabling theoretical maximum densification at low temperature. The insoluble two components effectively suppressed the grain growth by mutual pinning. The engineering implication of the developed YM nanocomposite imparts enhanced mechanical reliability, better cost effectiveness with excellent plasma resistance property over their counterparts in plasma using semiconductor applications.

## Introduction

Plasma processes are widely used for the fabrication of state-of-the-art highly integrated semiconductor circuits^1^. At present, high plasma power are inevitably required to control the dimensions of high-aspect-ratio contacts and deep-trench structures accurately (like in DRAM memory)^1,2^. Ceramic components like focus rings and shower heads in a plasma-etching chamber are easily exposed to high-power plasma and generate contamination particles that cause a decrease in the integrated circuit (IC) manufacturing yield^3–5^.In order to protect the IC from particle contamination, ceramic coating is applied to the interior of the plasma chamber. Most commercially adopted plasma resistant materials are sintered oxide ceramics such as Al_2_O_3_, quartz, and Y_2_O_3_^6–10^. Their high-temperature stability and chemical inertness found to be crucial in plasma shielding. Y_2_O_3_ is particularly more favorable than Al_2_O_3_^11–13^ due to its low etching rate and high chemical stability against CF_4_ plasma erosion^8,14^. However, Y_2_O_3_ ceramic suffers from unnecessary grain growth at high temperature^15^, making Y_2_O_3_ a challenging system to obtain grain size in the sub-micron range—the coarser grain size results in poor mechanical property. The plasma resistance in ceramic is associated with the porosity, roughness, defect density, and mechanical property^8,16^. Therefore, it is imperative to develop a system having a near theoretical density, fine grain size, low roughness, and high strength. Recently, Y_2_O_3_-MgO composites have been fabricated to tackle the above-mentioned issues with Y_2_O_3_ ceramics. The composite has better mechanical strength than their individual oxides as the presence of one phase impedes the grain growth of the adjacent phase due to the pinning effect at the grain boundary^17–19^. At the same time, yttria is a rare earth compound, making it an expensive plasma shielding candidate. On the other hand, magnesium oxide is comparatively cheaper, making the composite of Y_2_O_3_-MgO relatively economical with better plasma resistance property than the individual oxides.

To understand the erosion mechanism under CF_4_ and NF_3_ plasma, the erosion behavior of Y_2_O_3_, Al_2_O_3_ and YF_3_ have been reported^14,20,21^. An oxide surface exposed to fluorocarbon plasma was initially fluorinated and then removed by a physical process combined with thermal cycling in wafer processing equipment^21^.

It is notable that the surface roughness controlled by microstructures such as the grain size and defects such as pores for both bulk parts and coating parts strongly affect the amount of etching^16,22–24^. In an analogy to the referred reports, engineers in the semiconductor manufacturing field realized that proper control of the microstructure is the first and foremost requirement for high-performance structural ceramics^4^. In a recent study, etching rate was found to be dependent on the crystallographic orientation^25^. In other words, etching depth differs with different crystallographic plane for sintered polycrystalline ceramics. The authors are motivated by the above observation to determine the impact of grain anisotropic etching rate on ceramic surface roughness during plasma etching. Decrease in surface roughness eventually contributes in diminishing the particle generation problem during the plasma etching process^14^.

In this study, the plasma etching behavior of Y_2_O_3_·MgO composite ceramics (YM) featuring nano-sized grains was for the first time evaluated. A comparative study with the pore-free transparent Y_2_O_3_ and MgAl_2_O_4_ ceramics bas been carried out. In our previous report^26^, various transparent ceramics free from light-scattering pores, which also act as the origin and/or as a magnifier of the etching reaction, were proven to exhibit plasma resistance exceeding that of single-crystal sapphire. The nanostructured YM research has been pursued to its excellent mid-wavelength IR transmittance and reliable mechanical properties for use as hypersonic infrared windows, domes and eye-safe laser^27–31^. The plasma resistance of YM composite ceramics has not yet been studied, although the monolithic forms of both Y_2_O_3_ and MgO have demonstrated excellent plasma resistance^32^. In this work, the characteristics of the plasma etching behavior were mainly discussed in terms of the surface properties and microstructural aspects.

## Experimental procedure

Commercially available oxide powders of Y_2_O_3_ (99.9%, Rare Metallic, Japan), MgAl_2_O_4_ (99.9%, Baikowski, France) and MgO (99.99%, Sukgyung AT, Korea) were adopted as raw materials for the fabrication of monolithic Y_2_O_3_, monolithic MgAl_2_O_4_, and the composite YM ceramics. The details of the entire fabrication process of Y_2_O_3_ and MgAl_2_O_4_ are available in the literature^33,34^. The process to fabricate YM composite is first 50:50 vol% of Y_2_O_3_ and MgO powders were mixed via ball milling and dried to evaporate milling fluid. The powder mixtures are uni-axially pressed in steel mold and then the green compacts were sintered by hot-pressing at 1300℃ for 1 h under 40 MPa of pressure to reach nearly full theoretical density. After sintering, all samples were polished and masked on part of the top surface with polyimide tape to evaluate the etching properties. The thus-prepared nearly pore-free transparent ceramics were exposed to plasma using inductively coupled plasma. The details of the conditions used for plasma etching are listed in Table 1.

**Table 1:** Details of plasma etch conditions.

Parameters	Condition
Source power, W	500
Bias power, W	100
Pressure, mTorr	10
CF_4_:O_2_, SCCM	40:10
Etching time, h	1, 2, 3

The density of the ceramic samples was determined by the Archimedes method. The theoretical density of the Y_2_O_3_, MgAl_2_O_4_ and YM ceramics were 5.031, 3.58, and 4.30 g/cm^3^, respectively. The crystal structure of sintered specimens was identified by X-ray diffraction (XRD; D/Mas 2500, Rigaku, Japan). The etching depth was measured by a surface profiler (DXT-A, bruker, USA). The surface roughness and morphology were examined by the atomic force microscopy (XE-100, Park systems, Korea) and scanning electron microscopy (SU8230, Hitachi, Japan), respectively. The thermal diffusivity and heat capacity were measured by the laser flash analyzer (LFA 467, NETZSCH, Germany) and then the thermal conductivity (*k*) was calculated by following equation:

$$ k = \alpha \cdot \rho \cdot C_{p} $$

where *α*, *ρ* and *C*_*p*_ is the thermal diffusivity, density and heat capacity, respectively. The microhardness was measured by indentation method (HM, Mitutoyo, Japan) and the fracture toughness was determined by the Anstis Eq. ^35^.

## Results and discussion

**Figure 1:**
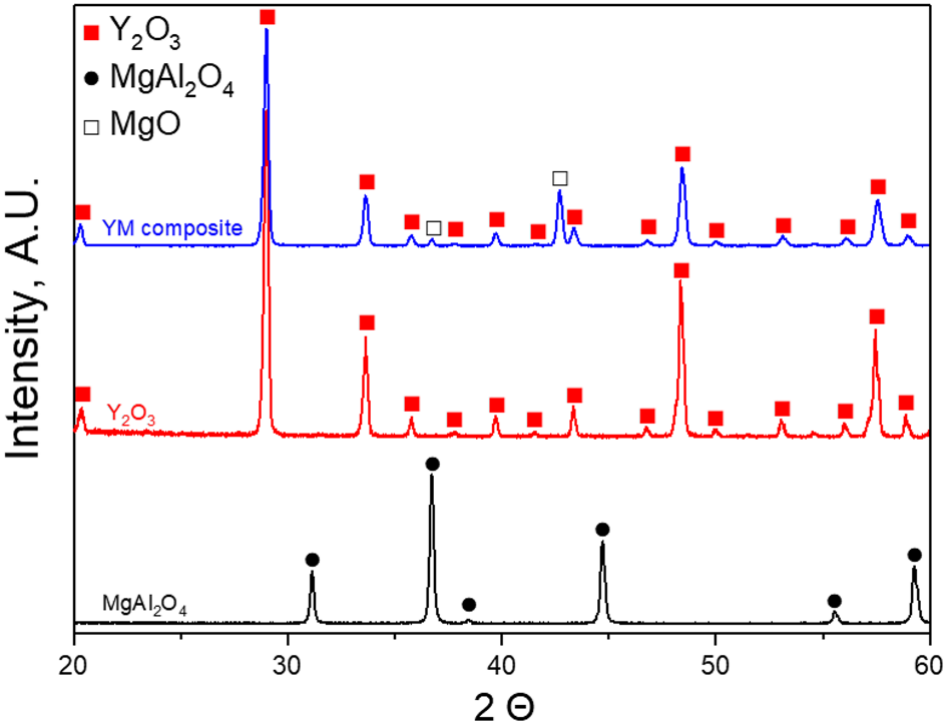
XRD patterns of YM composite, Y_2_O_3_ and MgAl_2_O_4_ ceramics.

Figure 1 shows the XRD patterns of sintered samples. The XRD peaks of YM composite have been indexed with the cubic Y2O3 (JCPDS 41-1105) and MgO (JCPDS 45-0946). No extra peak was noted in any sintered specimen, which confirms the phase purity of the samples.

**Figure 2:**
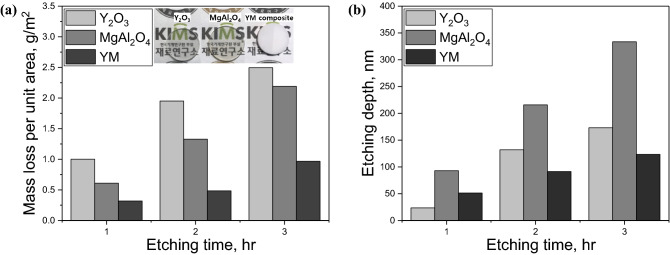
The measurement of (**a**) mass loss and (**b**) etching depth; the corresponding ceramic specimens after plasma etching are shown in the inset of Fig. (**a**) of which right half of each specimen was masked.

**Figure 3:**
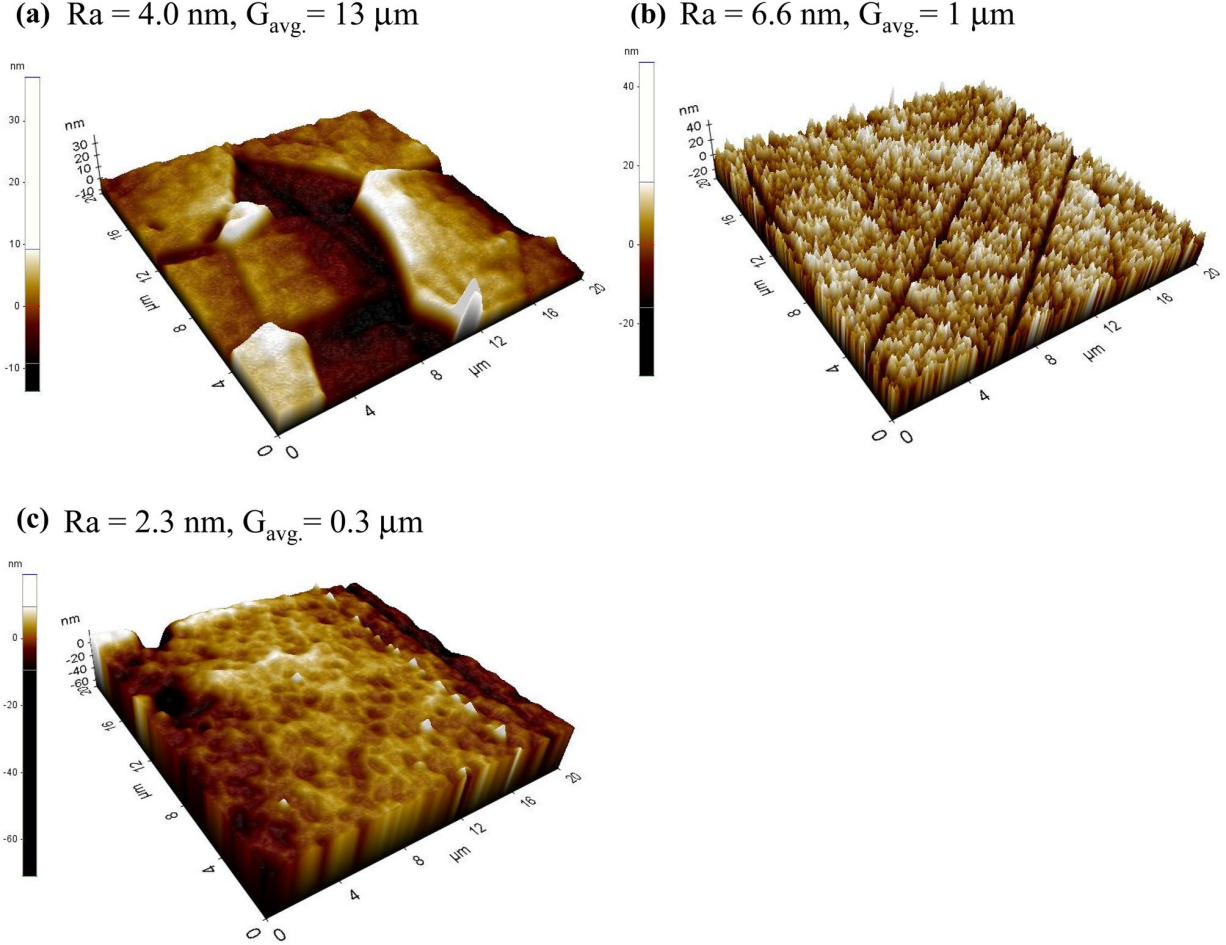
AFM images of plasma etched surface of (**a**) Y_2_O_3_, (**b**) MgAl_2_O_4_ and (**c**) YM.

**Figure 4:**
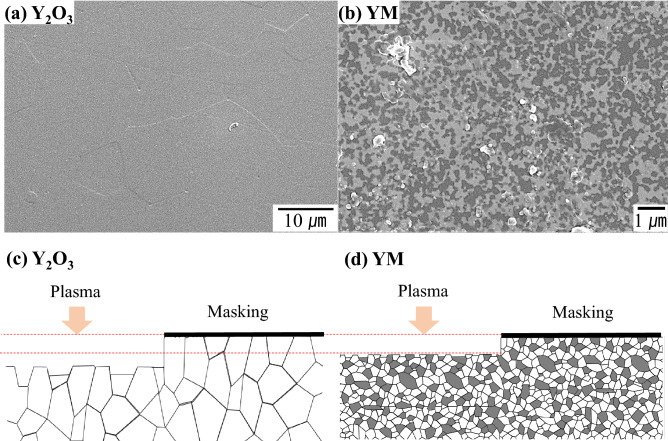
SEM and schematic images of plasma etched surface of (**a**, **c**) Y_2_O_3_ and (**b**, **d**) YM; (bright grain: Y_2_O_3_, dark grain: MgO).

The measured mass loss and etching depth of the ceramic specimens as a function of the plasma etching time are plotted in Fig. 2. With an increase in the plasma exposure time, both mass loss and etching depth monotonically increased for all specimens. The inversion of the magnitude of the mass loss and etching depth between Y_2_O_3_ and MgAl_2_O_4_ can be understood by considering the density differences, which were 5.031 g/cm^3^ for Y_2_O and 3.58 g/cm^3^ for MgAl_2_O_4_. Unlike the result of a medium level of mass loss, MgAl_2_O_4_ showed the deepest etching depth at all etching times. This tendency was noted to be similar to the earlier work^26^ and herein, based on the mass loss and etching depth with Y_2_O_3_ and MgAl_2_O_4_ ceramics, particle contamination caused by the Y_2_O_3_ ceramics is postulated to be more reducible than that by MgAl_2_O_4_ ceramics, but further work is necessary for sufficient clarification. It is known that the etching depth of sintered polycrystalline Y_2_O_3_ is roughly half that of single-crystal sapphire^20,32^, while a previous report by the authors using pore-free transparent Y_2_O_3_ found best-ever results with a record low 20% etching depth compared to that of sapphire^26^. It is surprising that the developed YM showed an even lower etching depth compared to that of transparent-grade Y_2_O_3_, which will be discussed later with the results of microstructural analysis of the etched surface morphology. The images in the inset of Fig. 2(a) were taken after plasma etching, where the right half of each specimen was masked. For visible wavelength both monolith Y_2_O_3_ and MgAl_2_O_4_ are transparent, while YM is opaque owing to scattering caused not by pores but mismatch in refractive index between Y_2_O_3_ and MgO. Meanwhile, the evaluation of plasma-resistance remains both unclear and controversial^4,36^. Accordingly, another way to characterize the etching behavior is to profile the etched. These results are presented in Figs. 3 and 4.

Figure 3 depicts AFM images of the etched surface profiling results. Topographical features after etching were clearly different in the three samples. The Y_2_O_3_ and YM ceramics tended to be homogeneously eroded by plasma exposure, resulting in an extremely smooth surface with R_a_ = 4.0 and 2.3 nm, respectively. On the other hand, the surface of MgAl_2_O_4_ was deeply eroded and, relatively rough; i.e., R_a_ = 6.6 nm. Generally, the surface roughness of conventional ceramics after plasma etching is reported to have a large range, from 100 nm to several microns, and these results have mainly been attributed to residual pores in the ceramics^4,8,20^. For transparent ceramics having nearly pore-free microstructures, smooth erosion (plateau-like surface morphology) was found to be possible without selective erosion caused by ion bombardment around the pores^4,25,26^. Given that the R_a_ value of the as-polished surface was under 2 nm, the AFM images in Fig. 3(c) reveal that the etched surface of YM was extremely smooth (R_a_ = 2.3 nm). Min et al. demonstrated that the surface roughness levels of transparent Y_2_O_3_, MgAl_2_O_4_ and sapphire were 9.0, 10.3 and 27.0 nm, respectively, under etching conditions identical to those in this study^26^. Kim et al. reported that the surface flatness during plasma etching played a critical role in reducing the generation of particles; it was noted that either the particles remained inside the chamber or were pumped out^21^. With an increase in the surface roughness, the condensed particle size simultaneously increased and the particles accumulated at the edge without being pumped out. The remaining large particles in the chamber may attach to the wafers, resulting in current shorts in integrated circuits^37^. From this point of view, YM ceramics yielding the most smoothly etched surface is expected to be used to create promising plasma-resistant materials, leading to reduced particulate contamination. Furthermore, in an atmosphere of fluorine-based plasma, the chemical reaction of Y_2_O_3_ and MgO are YF_3_ and MgF_2_, respectively. Their Gibbs free energy was estimated as -747 and -517 kJ/mol^38,39^. Therefore, a higher fluorine-plasma stability of MgO can explain a higher plasma resistance of YM composites than that of Y_2_O_3_ ceramics.

In order to elucidate the mechanism of the smooth surface roughness of the as-etched YM ceramics, the microstructural features of the Y_2_O_3_ and the YM ceramics were compared, as shown in Fig. 4. The grain sizes of the Y_2_O_3_ and YM were approximately 13 μm and 300 nm, respectively. Clearly, the etching depth of the coarser Y_2_O_3_ grain appears to differ depending on each grain, as shown in Fig. 4(a). This phenomenon can be explained by the different plasma etching behaviors with respect to the grain orientation in polycrystalline Y_2_O_3_ ceramics^25^. In contrast, the fine-grained microstructure of polycrystalline YM ceramics presented different etching behavior, as shown in Fig. 4(b). The schematics of the etched surface morphology in Fig. 4(c) and (d) specifically explain this. Based on the orientation-dependent erosion mechanism demonstrated by Kindelmann et al.^25^, each coarse Y_2_O_3_ grain started to erode specifically on certain crystal planes, such as the (001) or (011) plane, resulting in higher surface roughness and deeper etching depths relative to the fine-grained YM ceramics. That is, the etching speed is always rate-controlled by the subsequently emerging slow-etch-rate oriented Y_2_O_3_ grains upon the etching away of both the fast-etch-rate Y_2_O_3_ grains and all MgO upper grains. Therefore, the greater plasma resistance of YM over Y_2_O_3_ ceramics is primarily considered to originate from the effect of nano-sized grains, as smaller grain size is effective during exposure with a slow etch rate of Y_2_O_3_ grains on the surface. As for the monolith Y_2_O_3_ it is postulated that etch-rate might decrease with decreasing grain size by the same analogy. It should be emphasized that the theory of ceramic processing on making the composite between two insoluble components (Y_2_O_3_ and MgO) was successfully utilized to fabricate pore-free nano-sized grain microstructure.

Moreover, given that physical and chemical etching simultaneously occur during plasma etching^40^, another possible reason for the high plasma-resistance of YM ceramics is the higher mechanical reliability compared to Y_2_O_3_ and MgAl_2_O_4_^41^. Young’s modulus of materials which stands for the inherent physical property are in the order of MgAl_2_O_4_ > YM > Y_2_O_3_ as shown in Table 2. The results of mechanical and thermal properties of specimens indicate that the inherently inferior physical properties of Y_2_O_3_ ceramics can be overcame by adopting YM composite with the highest facture toughness and thermal conductivity. Kasashima et al. also reported a newly developed ceramic composite that showed enhanced plasma resistance given its improved mechanical properties and after an adjustment of the electrical resistivity^32^. The crucial microstructural aspects pertaining to the high strength of YM ceramics are the nano-sized Y_2_O_3_ and MgO grains, combining both suppressed grain growth and reaches full densification.

**Table 2:** Mechanical and thermal properties of specimens.

	Young's Modulus (GPa)	Microhardness (HV5, GPa)	Fracture Toughness (K_1C_, MPa∙m^1/2^)	Thermal conductivity (W/mK)
Y_2_O_3_	180^41^	7.6	2.0 ± 0.1	5.6
MgAl_2_O_4_	295^42^	14.0	2.5 ± 0.1	15.9
YM	230^41^	9.9	3.0 ± 0.3	15.3

## Conclusion

A nanocrystalline YM composite have been mainly studied to focus on its excellent IR transmittance and mechanical properties so far. The results here shed light for the first time on the feasibility of applying YM composite to parts requiring plasma resistance in the semiconductor processing industry. Specifically, the YM composite exhibited superior plasma resistance, including both a lower etching rate and smoother as-etched surface roughness relative of the transparent Y_2_O_3_ and Mg-spinel ceramic; originating from the effect of nano-sized grains with very carefully controlled microstructural defects, i.e., residual pores. Therefore, this novel trial with the YM composite for use in plasma environments is believed to set an important milestone to mitigate the particulate contamination issue in the semiconductor industry.
